# USING NATIONAL CORE INDICATORS—AGING AND DISABILITIES—FOR QUALITY, POLICY, AND SYSTEM TRANSFORMATION

**DOI:** 10.1093/geroni/igad104.0807

**Published:** 2023-12-21

**Authors:** Stephanie Giordano

**Affiliations:** Human Services Research Institute, Cambridge, Massachusetts, United States

## Abstract

National Core Indicators Aging and Disabilities (NCI-AD™), uses survey data to support State Medicaid, aging, and disability agencies to conduct surveys with individuals receiving publicly-funded long-term services and supports and measure and track their own outcomes and performance using reliable data collection methods and tools. Established in 2015, NCI-AD is now in use by 22 states nationwide. The NCI-AD portfolio includes the flagship survey – the Adult Consumer Survey (ACS) and the State of the Workforce—Aging and Disabilities (SotW-AD) survey. The ACS collects data directly from older adults and people with physical disabilities to assess the outcomes of services provided to individuals. Indicators address key areas of concern including service planning, rights, community inclusion, choice, health and care coordination, safety and relationships. In 2022, NCI-AD piloted the SotW-AD survey with five participating states. A first of its kind for the aging and disabilities field, this new effort collects and aggregates statewide information about wages, benefits, and turnover of the direct service workforce—as reported by the agencies that employ them. The SotW-AD will launch to all AD states in summer 2023. This session will provide an overview of the NCI-AD program, the type of data collected from each survey, and how state-level and research partners have utilized the data sets.

